# Self-organization of complex cortex-like wiring in a spiking neural network model

**DOI:** 10.1186/1471-2202-16-S1-P265

**Published:** 2015-12-18

**Authors:** Daniel Miner, Jochen Triesch

**Affiliations:** 1 Department of Neuroscience, Frankfurt Institute for Advanced Studies, Frankfurt am Main, Hessen 60486, Germany

## 

Understanding the structure and dynamics of cortical connectivity is vital to understanding cortical function. Experimental data strongly suggest that local recurrent connectivity in the cortex is significantly non-random, exhibiting above-chance bidirectionality, an overrepresentation of certain triangular motifs, and a heavy-tailed distribution of synaptic efficacies [1]. Additional evidence suggests a significant distance dependency to connectivity over a local scale of a few hundred microns [2], and particular patterns of synaptic turnover dynamics [3]. It is currently not understood how many of these non-random features arise. Gaining understanding, then, of the processes that lead to these complexities would provide valuable insights into the development and computational functionality of the cortex. While previous work has attempted to model some of the individual features of local cortical wiring, there is no model that comprehensively begins to account for all of them.

Here we present a spiking network model of a Layer V-like cortical slice culture (panel B) that, via the interactions of a few simple biologically motivated plasticity mechanisms, qualitatively reproduces many of these non-random effects, such as synaptic weight (panel A) and triangular motif distribution (panel C) Additionally, it reproduced experimentally observed synaptic growth and efficacy dynamics [3]. These plasticity mechanisms include spike timing dependent plasticity, synaptic normalization, homeostatic firing threshold adaptation, pruning of zero-efficacy synaptic connections, and the distance-dependent generation of new synaptic connections. As a spiking, topographic extension to the previously developed SORN family of models [4,5], there is also evidence suggesting that these plasticity mechanisms endow recurrent networks with powerful learning abilities. Our model suggests that mechanisms of self-organization arising from a small number of plasticity rules provide a parsimonious explanation for numerous experimentally observed non-random features of recurrent cortical wiring.

**Figure 1:**
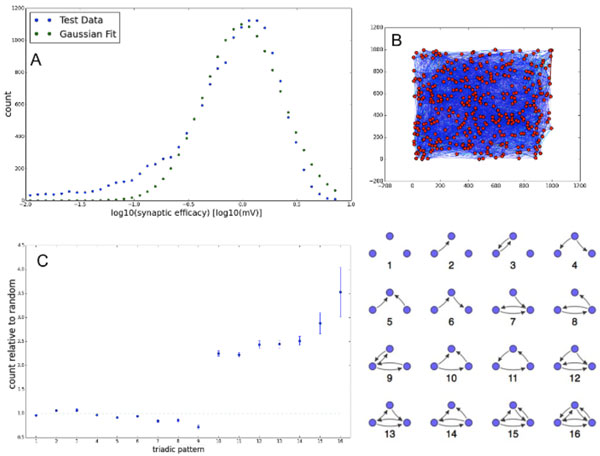
**Panel A: Mature synaptic weight distribution (in logarithmic space) for a simulation run with associated Gaussian (lognormal in linear space) fit**. Panel B: Topological graph of mature network for a simulation run. Panel C: Triangular motif count (relative to random and corrected for an overrepresentation of bidirectional connections, similar to [1]) for topological graph of mature network. Motif key to right.
